# A high-fibre personalised dietary advice given via a web tool reduces constipation complaints in adults

**DOI:** 10.1017/jns.2022.27

**Published:** 2022-04-28

**Authors:** Iris Rijnaarts, Nicole M. de Roos, Taojun Wang, Erwin G. Zoetendal, Jan Top, Marielle Timmer, Koen Hogenelst, Emily P. Bouwman, Ben Witteman, Nicole de Wit

**Affiliations:** 1 Division of Human Nutrition and Health, Wageningen University & Research, Stippeneng 4, 6708 WE Wageningen, The Netherlands; 2 Laboratory of Microbiology, Wageningen University & Research, Stippeneng 4, 6708 WE Wageningen, The Netherlands; 3 Wageningen Food and Biobased Research, Wageningen University & Research, Bornse Weilanden 9, 6708 PD Wageningen, The Netherlands; 4 Wageningen Economic Research, Wageningen University & Research, Bornse Weilanden 9, 6708 PD Wageningen, The Netherlands; 5 Department of Human Performance, The Netherlands Organization for Applied Scientific Research (TNO), Kampweg 55, 3769 DE Soesterberg, The Netherlands; 6 Gastroenterology and Hepatology Department, Hospital Gelderse Vallei, Willy Brandtlaan 10, 6716 RP Ede, The Netherlands

**Keywords:** Constipation, Dietary fibre, Functional bowel disorders, Personalised nutrition, Quality of life, BMI, body mass index, EMA, ecological momentary assessment, FFQ, food frequency questionnaire, IBS-C, Irritable Bowel Syndrome constipation predominant, MET, metabolic equivalent task, PAC-SYM, Patient Assessment of Constipation Symptoms, PAC-QoL, Patient Assessment of Constipation Quality of Life, PDA, personalised dietary advice, QoL, quality of life, SQUASH, short questionnaire to assess health-enhancing physical activity, VAS, visual analogue scale

## Abstract

Constipation can greatly impact the quality of life (QoL), which can be relieved by dietary fibres; however, preserving a higher fibre intake remains a challenge. We investigated the effects of a personalised dietary advice (PDA) on fibre intake and mild constipation complaints. A total number of twenty-five adults with mild constipation complaints were included in a 4-week observation period followed by a 4-week personalised intervention. The PDA provided high-fibre alternatives via a web tool. In weeks 1, 4 and 8, dietary intake, constipation complaints and QoL were assessed. Furthermore, participants collected a faecal sample at weeks 1, 4 and 8 to determine microbiota diversity and composition, and short-chain fatty acids (SCFA). Participants completed questions daily for 8 weeks regarding abdominal complaints, stool frequency and stool consistency. Fibre intake in week 8 was significantly higher compared to week 1 (*Δ* = 5·7 ± 6·7 g, *P* < 0·001) and week 4 (*Δ* = 5·2 ± 6·4 g, *P* < 0·001). Constipation severity and QoL significantly improved at week 8 compared to the observation period (*P* < 0·001). A higher fibre intake significantly reduced constipation severity (*β* = −0·031 (−0·05; −0·01), *P* = 0·001) and the QoL (*β* = −0·022 (−0·04; −0·01), *P* = 0·009). Stool consistency (*P* = 0·040) and abdominal pain (*P* = 0·030) improved significantly during the intervention period (*P* = 0·040), but stool frequency did not. Average microbial alpha diversity and composition and SCFA concentrations did not change over time, but indicated individual-specific dynamics. Several SCFAs were associated with constipation complaints. To conclude, a PDA effectively increased fibre intake and subsequently reduced constipation complaints, indicating that guided dietary adjustments are important and feasible in the treatment of mild constipation complaints.

## Introduction

Constipation complaints are characterized by straining, hard stools and infrequent bowel movements, which can greatly impact the quality of life (QoL)^(1)^. Moreover, constipation is associated with an increase of the risk of colorectal cancer, Parkinson's disease, cardiovascular disease and all-cause mortality among others^(2–7)^. The global prevalence is estimated between 5 and 20 % depending on the definition used and is more often present in women^(8–10)^. Constipation can result from having endocrine or metabolic disorders, neurological diseases, medication use or an unhealthy lifestyle^(11)^. A lifestyle characterized by a low-fibre intake and a low physical activity level is associated with an increased prevalence of constipation complaints^(12)^. Dietary fibres play an essential role in supporting a healthy stool pattern, as most fibres fasten intestinal transit time and absorb water, thus increasing intraluminal volume with a positive effect on stool frequency and stool consistency^(13–19)^. This was also shown in two meta-analyses, in which fibre supplements were effective in increasing stool frequency^(14)^, and inulin-type fructans improved a stool pattern^(20)^. Fibres can furthermore influence gut microbiota kinetics by fermentation of fibres into short-chain fatty acids (SCFA). Butyrate, one of the main SCFA, is a substrate for colonic cells and known for the anti-inflammatory properties and positive effects on gut health^(21–23)^. Furthermore, a high-fibre diet has been associated with higher levels of microbial richness and diversity^(24)^.

The effects of fibres from diet could also beneficially impact a stool pattern in adults with constipation complaints, but this is not fully researched yet. Anti *et al.* have shown that a fibre intake of >25 g/d increased stool frequency, which was more pronounced in patients who drank >2 l/day of water after an intervention of 2 months^(25)^. A high-fibre diet of 28 g/d was also effective in improving constipation in women with pelvic floor disorders after a 42-d intervention^(26)^. Moreover, a high-fibre diet improved the QoL of people with constipation, as was shown in elderly and patients with a chronic kidney disease^(27,28)^. Interestingly, medical costs associated with constipation complaints seem to reduce with an increased fibre intake^(29,30)^.

A fibre intake of 14 g/1000 kcal, which is 30 g/d for women and 40 g/d for men, is recommended for adults in the Netherlands, regardless of having constipation complaints^(31)^. However, median current intakes are far below these recommendations, as Dutch women consume 18 and men 23 g/d^(32)^. A personalised dietary advice (PDA) was recently suggested as a strategy to sustainably improve the diet with promising results^(33,34)^. The PDA improved compliance to a high-fibre, high-water diet in children with refractory functional constipation compared to general advice^(33)^. However, this study used a face-to-face guidance in their PDA, making it difficult to reach larger populations. In the Food4Me trial, a digital PDA was shown to be effective in improving healthy eating index scores, but not dietary fibre intake in 1607 healthy adults^(34)^. However, the study population had high baseline fibre intakes, and an increase in fibre was not the sole aim of the intervention. Recently, we have shown that a digital high-fibre PDA was effective in improving fibre intake up to 3 months after the intervention in adults without gastrointestinal complaints, and this PDA was positively evaluated^(35)^. Therefore, we now aimed to investigate the effect of a high-fibre PDA on constipation severity, QoL, stool pattern and fibre intake in adults with mild constipation complaints. Furthermore, the effects of a digital high-fibre PDA on faecal microbiota and SCFA, behavioural factors and acceptability were investigated.

## Methods

**Fig. 1.**
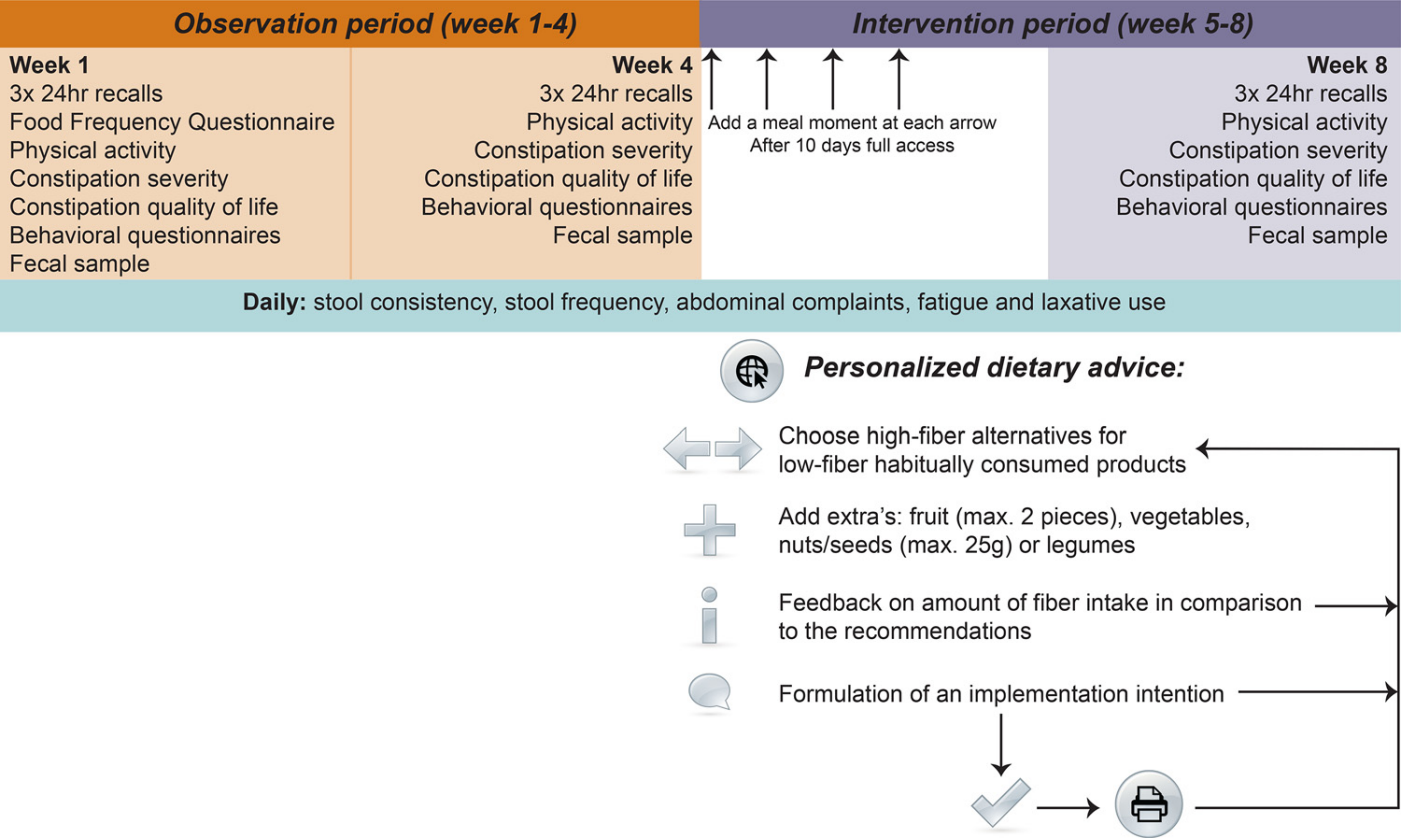
Study design.

This study had an 8-week study period consisting of one arm. The study consisted of two phases. The first phase was a 4-week observation period (weeks 1–4), in order to take the high within- and between-person variability in a stool pattern, complaints and dietary intake into account^(36,37)^ and to serve as a control. Thereafter, a 4-week intervention period followed (weeks 5–8), in which participants received the PDA (Fig. 1). To reduce bias, participants were unaware of the purpose of the PDA during the observation period, e.g. they were informed that the intervention would include lifestyle advice but not that it was focused on fibre. At the start of the intervention, participants received this information. The study was performed from August to November 2020. This study was conducted according to the guidelines laid down in the Declaration of Helsinki, and all procedures were approved by the Medical Ethics Committee of Brabant (P2013). All participants provided written informed consent. The study was registered at Clinicaltrials.gov under number NCT04457791 (https://clinicaltrials.gov/ct2/show/NCT04457791).

### The PDA intervention

As described earlier^(35)^, the PDA was distributed via a web tool developed for this study and was generated by linking personal food intake to generic food data. The PDA aimed to provide high-fibre substitutes for habitually consumed low-fibre products. The advice was personalised based on gender and habitual dietary intake of the last month, as assessed by a 247-item meal-based food frequency questionnaire (FFQ). The FFQ was performed in week 1 during a face-to-face interview with trained researchers. The FFQ was validated^(38,39)^, except that items were not questioned for the whole day but per meal moment (breakfast, lunch, dinner and in-between meals), so that advices could be given per meal moment.

The web tool showed a participants’ habitual intake per meal moment (breakfast, lunch, dinner and in-between meals) and high-fibre alternatives, which were ranked from high to low based on fibre content, to aid participants in their selection of high-fibre alternatives. The high-fibre alternative list did not use brand names but generic product categories (for example, whole wheat crackers) and was compiled by study researchers in consultation with dieticians. Participants could also include an extra portion of fruit, vegetables, legumes and/or nuts and seeds at each meal moment. In line with the Dutch recommendations, participants could not select >2 pieces of fruit and >25 g of nuts and seeds per day^(40)^ to limit sugar and calorie intake. Participants then received feedback on how much their chosen PDA increased their daily dietary fibre intake in reference with the recommendations. The final step included the formulation of implementation intentions, which can help participants to translate their intentions into behaviour and achieve sustainable dietary changes into the daily routine^(41)^.

During the first 10 d of the intervention, participants were limited in their selection of meal moments to ensure a gradual increase in dietary fibre intake to prevent abdominal bloating or cramps. From day 1 to 3, they could select one meal moment to work on, on days 4–6 they added a second meal moment to their PDA, and so on. After 10 d, participants had access to all meal moments in the PDA, and they could freely adjust their PDA during the remainder of the intervention (Fig. 1). The web tool also stated general lifestyle tips regarding water intake and physical activity^(42)^, and information on how to read food labels. Participants’ activity on the web tool was logged to assess compliance.

### Study participants

Participants were recruited via the participant database of Wageningen University & Research, social media and newspaper advertisements. Participants were eligible when having mild constipation complaints, which were defined as being unsatisfied with their stool pattern (<6 on a visual analogue scale (VAS) from 1 to 10), and habitual stool form of Bristol stool type 1–4^(43)^ and/or ≤4 defecations per week. These criteria are less stringent than the official functional constipation definition yet were chosen for several reasons.

First, although the Rome IV criteria for constipation are validated, studies have shown a large overlap with an Irritable Bowel Syndrome constipation subtype (IBS-C), and current diagnostics are unable to distinguish between both disorders^(11,12)^. Second, 19–34 % of the people who experience constipation complaints do not meet the Rome criteria for constipation or IBS-C but still experience substantial symptoms and a reduction in the QoL^(13–15)^, and are frequently missed in research and treatments. Third, we expected that a mildly constipated population can benefit the most from a dietary fibre intervention; hence, the main inclusion criteria were based on stool satisfaction, in combination with either a hard or normal Bristol stool type and/or a low stool frequency. Frequent loose stools and diarrhoea were excluded. Last, self-evaluation of constipation complaints using the VAS and the Bristol stool type was shown useful to determine constipation^(44)^.

Other criteria included a restriction of age to 18–55 years and body mass index (BMI) of <30 kg/m^2^ due to national restrictions because of the Sars-CoV-2 pandemic. Furthermore, eligible participants were living near the city of Wageningen (maximum of 50 km) for practical reasons, had a relatively low-fibre intake (females <26 g/d, males <33 g/d), and in possession of and able to use a computer and mobile phone. Participants were excluded when having an autonomic disorder, inflammatory bowel disease, coeliac disease, cancer, kidney disease, depression or hypothyroidism; when following a diet and unable or unwilling to change; pregnant or breastfeeding; using diuretics, antidepressants, codeine, antibiotics, fibre supplements, such as prucalopride, methylnaltrexone or linaclotide.

We aimed to include twenty-five participants in the intervention period to measure an increase in stool frequency of 1·3  (1·8) stools/week with *α* = 0·05 and 1 − *β* = 0·80^(25)^. We screened participants for a low-fibre intake in two steps: first, a rough screening was done by using our specially developed screening fibre questionnaire^(45)^. Next, a second and more thorough screening based on a complete FFQ was performed. As we expected that 20 % of the screened participants would have a fibre intake exceeding the cut-offs, we included thirty participants to complete the FFQ, to result with twenty-five participants below the cut-offs in the intervention phase.

### Constipation complaints and stool pattern

Constipation severity, QoL and stool pattern were the primary outcomes. Constipation severity of the last 2 weeks was assessed by using the 12-item validated Patient Assessment of Constipation Symptoms (PAC-SYM)^(46,47)^. This questionnaire gives a score for total severity and severity subscales for abdominal pain, stool complaints and rectal complaints. Each score ranges from 0 to 4, with a high score indicating severe symptoms. The validated 28-item Patient Assessment of Constipation QoL (PAC-QOL) was used to assess the impact of constipation on daily life during the last 2 weeks^(48)^. This questionnaire computes a score for total QoL and subscale scores for worries and concerns, satisfaction of stool pattern, physical discomfort and psychological discomfort. Scores range from 0 to 4, a high score indicating a poor QoL. Both questionnaires were completed digitally in weeks 1, 4 and 8.

Abdominal complaints, stool pattern and laxative use were assessed daily during the 8-week study period by using an Ecological Momentary Assessment (EMA) app on participants’ mobile phone. The EMA is a structured diary technique that can take personal variation into account^(49)^ and has previously been used to assess a stool pattern in IBS patients^(50)^. In the present study, participants received notifications every evening (time could be personalised), and questions could be answered within 1 h after the notification. Participants rated abdominal cramps, pain, bloating, flatulence and fatigue on a 100-point VAS from ‘no complaints/fatigue’ to ‘very severe complaints/fatigue’^(51,52)^. Moreover, participants reported laxative use, stool frequency as well as stool consistency by using the Bristol stool chart, which lists stools from small pallets (type 1) to very loose (type 7)^(43)^.

### Dietary intake and physical activity

To assess changes in fibre intake and diet between weeks 1, 4 and 8, trained research dieticians performed 24-h recalls via the telephone. For each timepoint, three non-consecutive recalls consisting of 2 weekdays and 1 weekend day were performed to take variation into account. Participants were not informed beforehand which day the recall would take place to reduce bias. Recalls were subsequently entered in the validated programme Compl-eat^(53)^, which estimated nutrient intake by using the Dutch Food Composition Table of 2019^(54)^. Furthermore, high-fibre food group intake was compiled from the 24-h recall data and included whole grain bread/crispbreads, whole grain cereals and grains (e.g. rice, pasta and couscous), vegetables, fruits, nuts and seeds, legumes, and potatoes and other tubers. Subjective self-efficacy of eating more fibre was reported daily during the 4-week intervention via the EMA app. Participants completed the question ‘did you manage to eat more fiber today’ on a 100-point VAS ranging from 0 ‘not at all’ to 100 ‘yes, very much’.

Physical activity was assessed at weeks 1, 4 and 8 by using the validated short questionnaire to assess health-enhancing physical activity (SQUASH)^(55)^. This questionnaire assessed commuting, leisure time, sports, household and work/school activities. For each activity, a score was calculated by multiplying the metabolic equivalent of task (MET) values, derived from the Ainsworth compendium^(56)^, by the duration of the activity. Furthermore, a total activity score was computed by summing the score of all activities.

### Faecal microbiota and SCFA profiling

Participants collected a faecal sample in weeks 1, 4 and 8 of the study. The sample was immediately frozen at home, and participants transported the frozen sample to the research facility within 7 d by using a dedicated cooling box. Subsequently, the sample was put on dry ice and stored at the −80°C freezer until further analysis.

Faecal SCFA acetate, propionate and butyrate were analysed as previously described, with minor modifications^(57)^. Briefly, 0·4 g of faeces was used, and mixed thoroughly with 1·6 ml demi water to extract the SCFA, which were analysed by High-Performance Liquid Chromatography (HPLC, LC-2030C, Shimazu, Kyoto, Japan) with a Shodex SH1821 column (Showa Denko K.K., Tokyo, Japan). Microbiota composition was determined as previously described^(58)^. In short, 0·25 g of faeces (wet weight) was used for DNA isolation with the repeated beating method^(59)^. Subsequently, PCR amplification of the V4 region of the 16s rRNA gene followed by the barcoded Illumina Hiseq2500 sequencing (150 bp paired end) was performed to obtain sequencing data^(60)^. Afterwards, NG-Tax 2·0 was used to process the raw sequencing data for Amplicon Sequencing Variant (ASV) picking with default settings and for taxonomic assignments by using the SILVA database (version 132)^(61,62)^. Sequencing data were submitted to the European Nucleotide Archive with accession number PRJEB47379.

### Behavioural and PDA evaluation questionnaires

Validated behavioural questionnaires were completed to gain insights into how the PDA affected the participants and why the PDA was effective or not. In weeks 1, 4 and 8, participants filled in a 3-item intention to eat fibres, a 2-item subjective health and a 5-item self-regulation questionnaire^(63,64)^. At weeks 4 and 8, participants completed a 5-item subjective knowledge and a 9-item outcome belief questionnaire regarding fibres^(65,66)^. Answers were rated on a 7-point Likert scale. When filling in these questionnaires, participants were blinded for fibre in week 1, but not in weeks 4 and 8. Participants also received an evaluation questionnaire in week 8 to assess acceptance of the PDA. Participants rated statements on a 7-point Likert scale, which included how positive, useful, attractive or interesting they found the advice, and how much the PDA helped and/or motivated them.

### Statistical analysis

Continuous data are presented as mean  (standard deviation), or median (interquartile range, IQR) when skewed. Differences over time (fixed main factor) in symptoms, QoL, diet, physical activity and SCFA were assessed using mixed models with a diagonal structure. Furthermore, mixed models were used to assess the effects of fibre intake (main fixed covariate) on constipation severity or QoL (dependent variables). In an additional model, water intake and total physical activity score were added to assess the effects of fibre when these variables were adjusted for. Mixed model data are reported as the *β* coefficient with 95 % confidence intervals or the standard error. Based on the minimal important difference (MID) of total PAC-SYM, a change of ≥0·6 was considered clinically relevant^(67)^, and responders and non-responders to the intervention period were defined and compared with an independent sample *t*-test. To analyse EMA data (stool pattern and abdominal complaints), linear mixed models with the restricted maximum likelihood estimation using lmer were used. Participants that completed ≥40 out of 56 d for EMA questionnaires were included in EMA analysis. The behavioural questionnaires were analysed by using the general linear model with repeated measures.

Microbiota alpha diversity (within sample diversity) and composition were calculated at the ASV level by using Phyloseq^(68)^. ASV richness and Shannon diversity were calculated for assessing microbiota alpha diversity, which were compared between timepoints by using a Wilcoxon signed-rank test. Principle coordinate analysis (PCoA) based on unweighted (considering the presence/absence of ASVs) and weighted (considering ASVs and their relative abundance) Unifrac distances^(69)^ was performed for the visualisation of microbiota composition.

For the microbiota data, *P*-values for multiple pairwise tests were corrected by using the Benjamini–Hochberg false-discovery rate. Microbiota and EMA data were analysed in R version 4·0·0^(70)^, and other data in SPSS version 25 (IBM Corp., Armonk, NY, USA). A (corrected) *P*-value of ≤0·05 was considered significant.

## Results

**Fig. 2.**
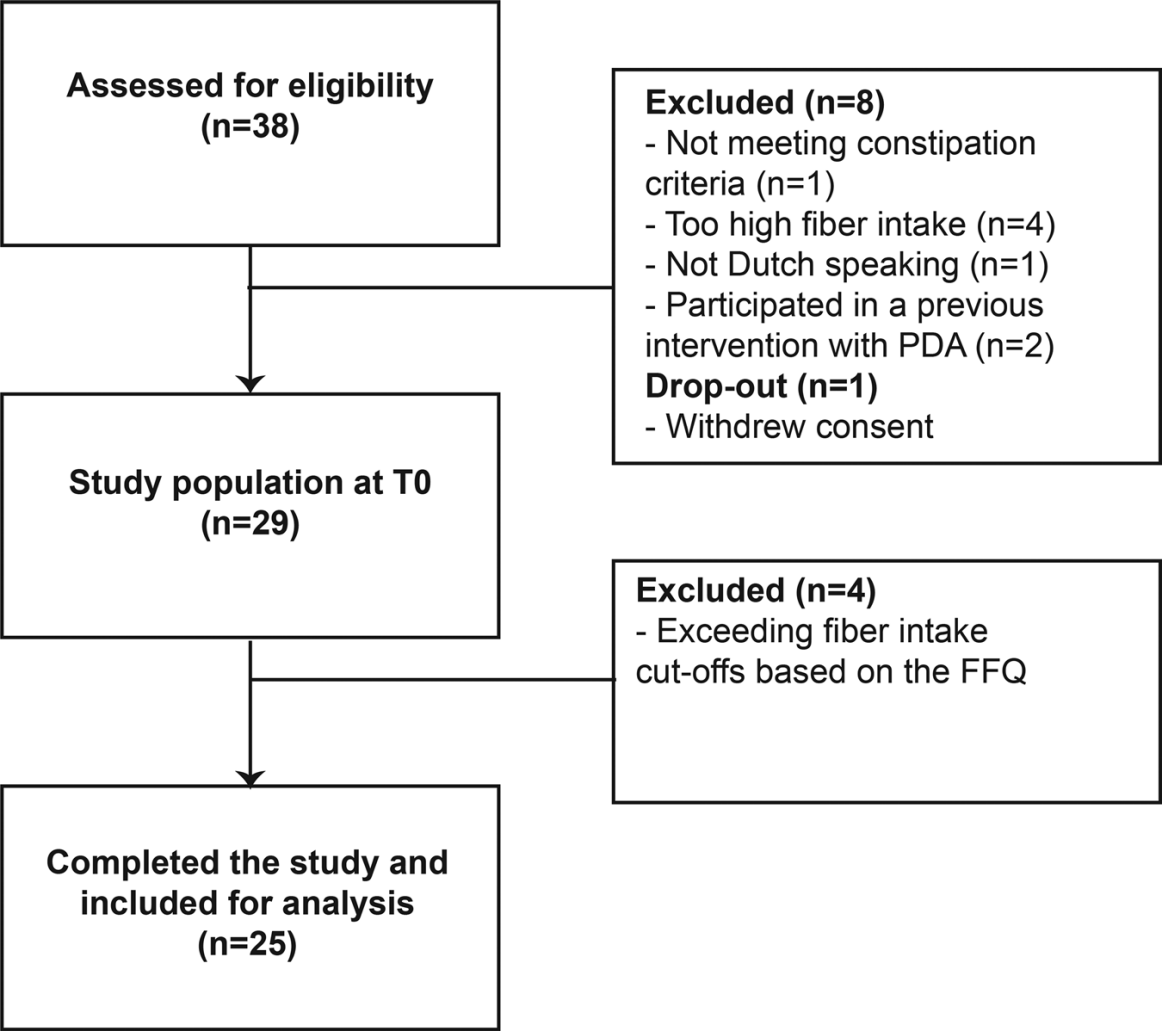
Study flowchart.

In total, thirty-eight participants were screened, one participant withdrew consent before study start, and twenty-nine participants were included in the study (Fig. 2). Four participants were excluded in week 3 in line with the study protocol, resulting in twenty-five participants as the final study population. The study population consisted mainly of young, female participants with a higher education level (Table 1). None were currently smoking nor used laxatives at the start of the study. All participants logged in on the PDA web tool at least once and, on average, completed all steps on the web tool 3·7  (2·2) times during the 4-week intervention. Fruit was added to the PDA most frequently (*n* 14), followed by vegetables (*n* 10), nuts and seeds (*n* 8) and then legumes (*n* 7).

**Table 1.** Baseline characteristics of the study population

	Constipated adults (*n* 25)	
Age (years)	26	23–53
Gender, males *n* (%)	5	20
BMI (kg/m^2^)	23	2·3
Completed ≥ higher vocational education, *n* (%)	20	80
Satisfaction with a stool pattern^a^	3·1	1·5
Stool frequency (number of stools/week)	4·2	1·8
Habitual stool type^b^	2·7	1·0

Values are mean and standard deviations or median (interquartile range) when skewed.

a Assessed on a VAS from 1 ‘not satisfied’ to 10 ‘very satisfied’.

b Indicated by the Bristol stool chart, which rates stools from small pallets (type 1) to very loose (type 7).

### PDA increased fibre intake, while other lifestyle parameters stayed stable over time

Dietary fibre intake, both in grams and g/1000 kcal, was significantly higher in week 8 compared to week 1 (*Δ* = 5·7 (6·7) g, *P* < 0·001 and *Δ* = 1·5  (3·2) g/1000 kcal, *P* = 0·032) and week 4 (*Δ* = 5·1  (6·4) g, *P* < 0·001 and *Δ* = 1·9  (3·2) g/1000 kcal, *P* = 0·007, Table 2), indicating that the increase in fibre intake was specifically during the intervention period. Furthermore, the percentage of participants adhering to the recommendations of fibre increased over time, with statistical significance for fibre in grams (12–36 %, *P* = 0·023), but not for g/1000 kcal (16–40 %, *P* = 0·148). Self-reported self-efficacy of increasing fibre intake was significantly lower during the weekend compared to weekdays (*P* = 0·004, Supplementary Figure S1). Participants significantly increased the amount of fibre from whole grain breads (*P* = 0·011) and fruit (*P* = 0·031) at week 8 compared to the observation period, but not from whole grain cereal and grains (*P* = 0·755), vegetables (*P* = 0·537) and potatoes (*P* = 0·370, Supplementary Figure S2). The fibre content from nuts and seeds (*Δ* = 0·69 (1·7) g/fibre, *P* = 0·163) and legumes (*Δ* = 0·98  (3·4) g/fibre, *P* = 0·085) increased after the PDA, albeit non-significantly. During the 8-week study period, physical activity, bodyweight, energy, water and macronutrient intake remained stable (Supplementary Table S1).

**Table 2.** Efficacy of the intervention and changes in lifestyle over time

	Week 1		Week 4		Week 8		*P*-value
Efficacy of the intervention: dietary fibre intake							
Dietary fibre (g)	21·6^a^	7·1	21·0^a^	6·7	26·7^b^	9·8	**0·025**
Adhering to fibre recommendation in grams, *n* (%)^†^	3	12	2	8	9	36	**0·023**
Dietary fibre (g/1000 kcal)	11·2^a^	2·9	11·6^a^	3·2	13·1^b^	3·9	**0·022**
Adhering to fibre recommendation per 1000 kcal, *n* (%)^†^	4	16	6	24	10	40	0·148
Dietary intake							
Energy (kcal)	1938·2	462	1848·7	446	2044·7	444	0·305
Carbohydrates (en%)	42·2	5·9	44·8	5·7	43·2	6·2	0·275
Water (litre)^‡^	2·74	2·4–3·5	2·57	2·3–3·0	2·8	2·8–3·2	0·829
Physical activity							
Total physical activity score*	5700	2490–7478	5865	4510–7080	4530	3190–6525	0·271
Adhering to the recommendation, *n* (%)^†^	14	56	13	52	14	56	0·948

Values are mean and standard deviations or median (interquartile range) when skewed. Dietary intake was assessed using 24-h recalls and physical activity using the short questionnaire to assess health-enhance physical activity (SQUASH). Differences between timepoints were assessed using linear mixed models or *χ*^2^ when categorical, different superscripts indicate significant differences between the timepoints. The overall *P*-value over time is shown, significance is indicated in the bold text. Abbreviations: en% = energy percentage.

† Recommendations for fibre are according to the Dutch Health council; 30 g for women or 40 g for men, or 14 g/1000 kcal. The physical activity guideline is >30 minutes of moderate or vigorous physical activity for ≥5 days/week.

‡ Water intake not only represents intake of liquids but also includes water in foods.

* Calculated by multiplying the metabolic equivalent of task values per activity times the minutes per week per activity, and then summed. *P*-values <0.05 were considered significant and indicated in bold.

### Dietary fibre intake significantly improved constipation complaints over time

**Fig. 3.**
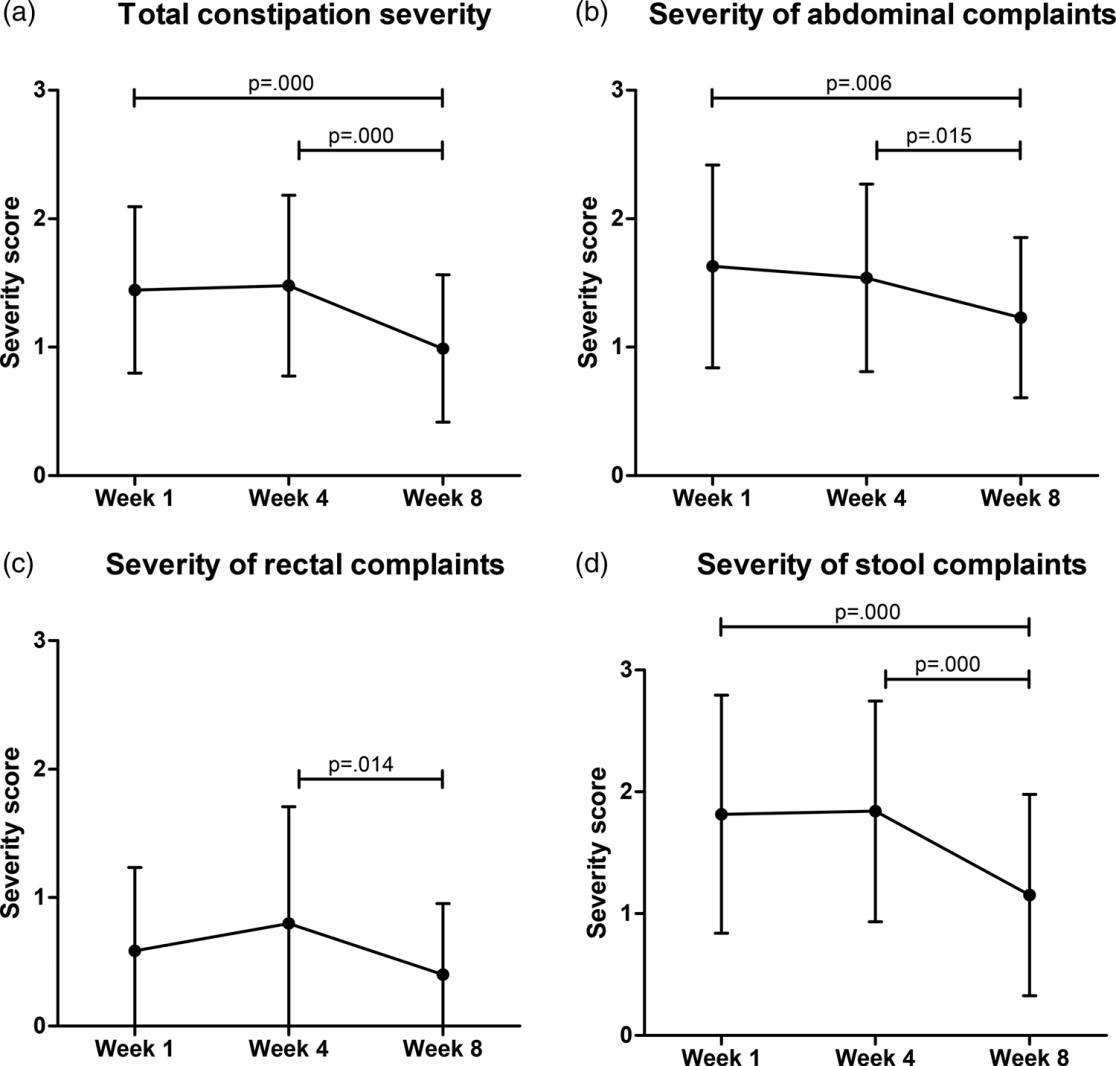
Changes in constipation severity over time. **Legend:** measured by the PAC-SYM questionnaire. Scores range from 0 to 4, a higher score indicating more severe constipation. Differences over time were tested with linear mixed models. Weeks 1 and 4 were observational, and week 8 is after the intervention.

**Fig. 4.**
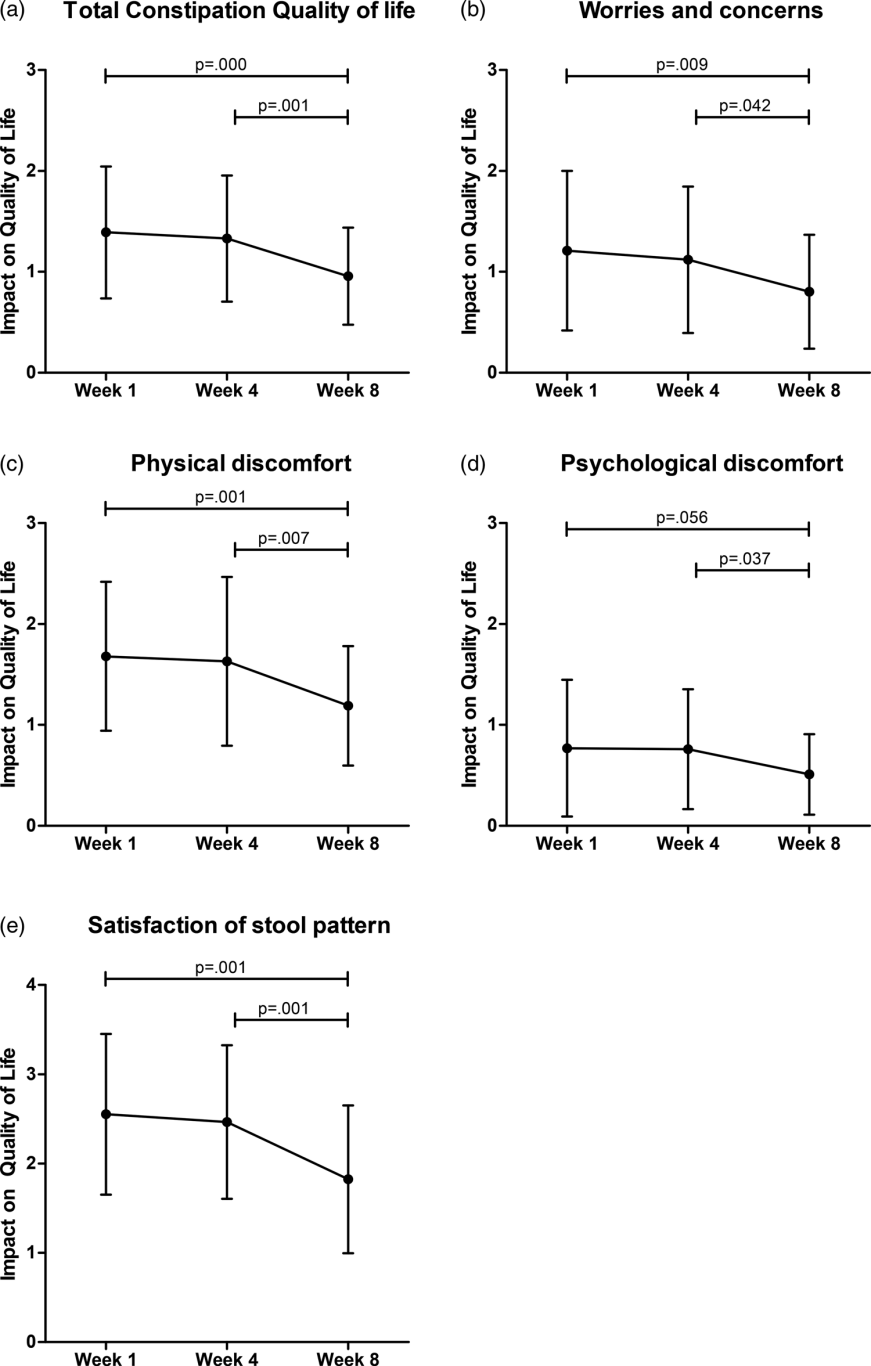
Changes in the constipation-related QoL over time. l**Legend**: measured by the PAC-QoL questionnaire. Scores range from 0 to 4, a lower score indicating a better QoL. Differences over time were tested with linear mixed models. Weeks 1 and 4 were observational, and week 8 is after the intervention.

Total constipation severity (scored from 0 to 4) improved significantly at week 8 compared to the observation period (week 1 = 1·49  (0·6), week 4 = 1·48  (0·7), week 8 = 0·99  (0·6), *P* < 0·001, Fig. 3(a)). Similar results were found for its subscales abdominal complaints (*P* = 0·003, Fig. 3(b)) and stool complaints (*P* < 0·001, Fig. 3(d)). Although rectal complaints did significantly change over time (*P* = 0·017, Fig. 3(c)), pairwise comparison showed that this was only between week 4 and week 8 (*P* = 0·014). The total constipation QoL improved significantly over time (*P* = 0·001, Fig. 4(a)), as well as worries and concerns (*P* = 0·014, Fig. 4(b)), physical discomfort (*P* < 0·001, Fig. 4(c)) and stool satisfaction (*P* < 0·001, Fig. 4(e)). Psychological discomfort did not change significantly over time (*P* = 0·053, Fig. 4(d)).

Mixed model analysis showed that fibre intake (g/d) significantly affected all scores of constipation severity and QoL over time, except for psychological discomfort (*β* = −0·013  (0·008), *P* = 0·121, Table 3). This indicates that the change in constipation severity or the QoL score was dependent on dietary fibre intake over time. Results did not change after the addition of water intake and physical activity level to the model.

**Table 3.** Mixed model analysis of the effects of fibre intake on constipation severity and the QoL over time

	Model 1: fibre intake			Model 2: fibre, water and physical activity score		
	Estimate	95 % CI	*P*-value	Estimate	95 % CI	*P*-value
Total constipation severity	−0·031	−0·05, −0·01	**0·001**	−0·028	−0·05, −0·01	**0·003**
Abdominal complaints	−0·027	−0·04, −0·01	**0·004**	−0·024	−0·04, −0·00	**0·014**
Rectal complaints	−0·021	−0·04, −0·00	**0·021**	−0·021	−0·04, −0·00	**0·028**
Stool complaints	−0·038	−0·06, −0·01	**0·004**	−0·036	−0·06, −0·01	**0·008**
Total constipation quality of life	−0·022	−0·04, −0·01	**0·009**	−0·021	−0·04, −0·00	**0·013**
Worries and concerns	−0·022	−0·04, −0·00	**0·026**	−0·023	−0·04, −0·00	**0·024**
Satisfaction of stool pattern	−0·041	−0·07, −0·01	**0·003**	−0·031	−0·06, −0·00	**0·022**
Physical discomfort	−0·033	−0·05, −0·01	**0·002**	−0·033	−0·05, −0·01	**0·003**
Psychological discomfort	−0·013	−0·03, 0·00	0·121	−0·014	−0·03, 0·00	0·075

The estimate and *P*-value are given for fibre intake in grams. Data are tested using linear mixed models, using a diagonal variance structure and indicating time as repeated measures. Constipation severities in the QoL are dependent variables and lifestyle variables are added as fixed main effects to the model. Dietary intake was assessed using 24-h recalls, and physical activity using the short questionnaire to assess health-enhance physical activity (SQUASH). Physical activity is a score calculated by multiplying the metabolic equivalent of task values per activity times the minutes per week per activity, and then summed. *P*-values <0.05 were considered significant and indicated in bold.

### Stool consistency and abdominal pain improved, but not stool frequency

**Fig. 5.**
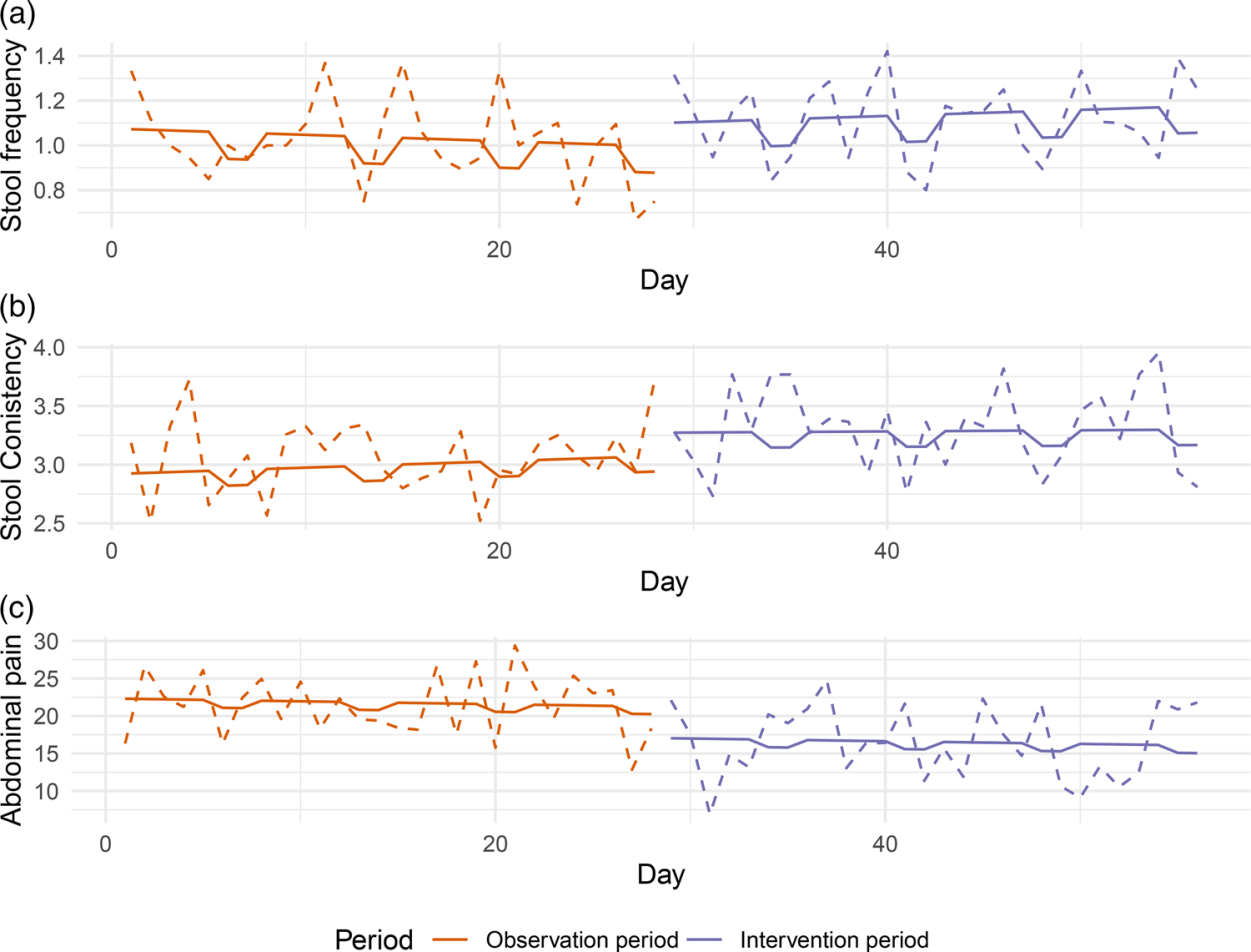
Analysis of daily measurements of stool pattern and complaints over time **Legend:** data were collected daily using the EMA application on a participants’ mobile phone. The dotted line represents the group average, the solid line represents the regression line. (a) Stool frequency per day, 0 indicating no stool that day. (b) Stool consistency, assessed by the Bristol stool chart per day, ranging from 1 ‘hard pellets’ to 7 ‘loose stools’. (c) Abdominal complaints assessed on a 100-point VAS from 0 ‘no complaints’ to 100 ‘very severe’.

EMA compliance was high: 85  (14) % of the questionnaires were completed. None of the participants reported the use of laxatives during the 8-week trial. Four participants did not complete ≥40/56 days, resulting in twenty-one participants as the study population for analysis. There was no intervention effect on the average number of stools per day (*P* = 0·795, Fig. 5(a)), but stool consistency significantly softened during the intervention period (3·2 (95 % CI = 2·9, 3·6)) compared to the observation period (2·9 (95 % CI = 2·6, 3·3), *P* = 0·041, Fig. 5(b)). Furthermore, abdominal pain significantly reduced during the intervention period (16·0 (95 % CI = 8·7, 23·3)) compared to the observation period (21·3 (95 % CI = 14·0, 28·6), *P* = 0·03, Fig. 5(c)). No intervention effects were observed for fatigue (*P* = 0·238), abdominal cramps (*P* = 0·331) or bloating (*P* = 0·136), results not shown.

### Faecal gut microbiota and SCFA do not change after the intervention period

**Fig. 6.**
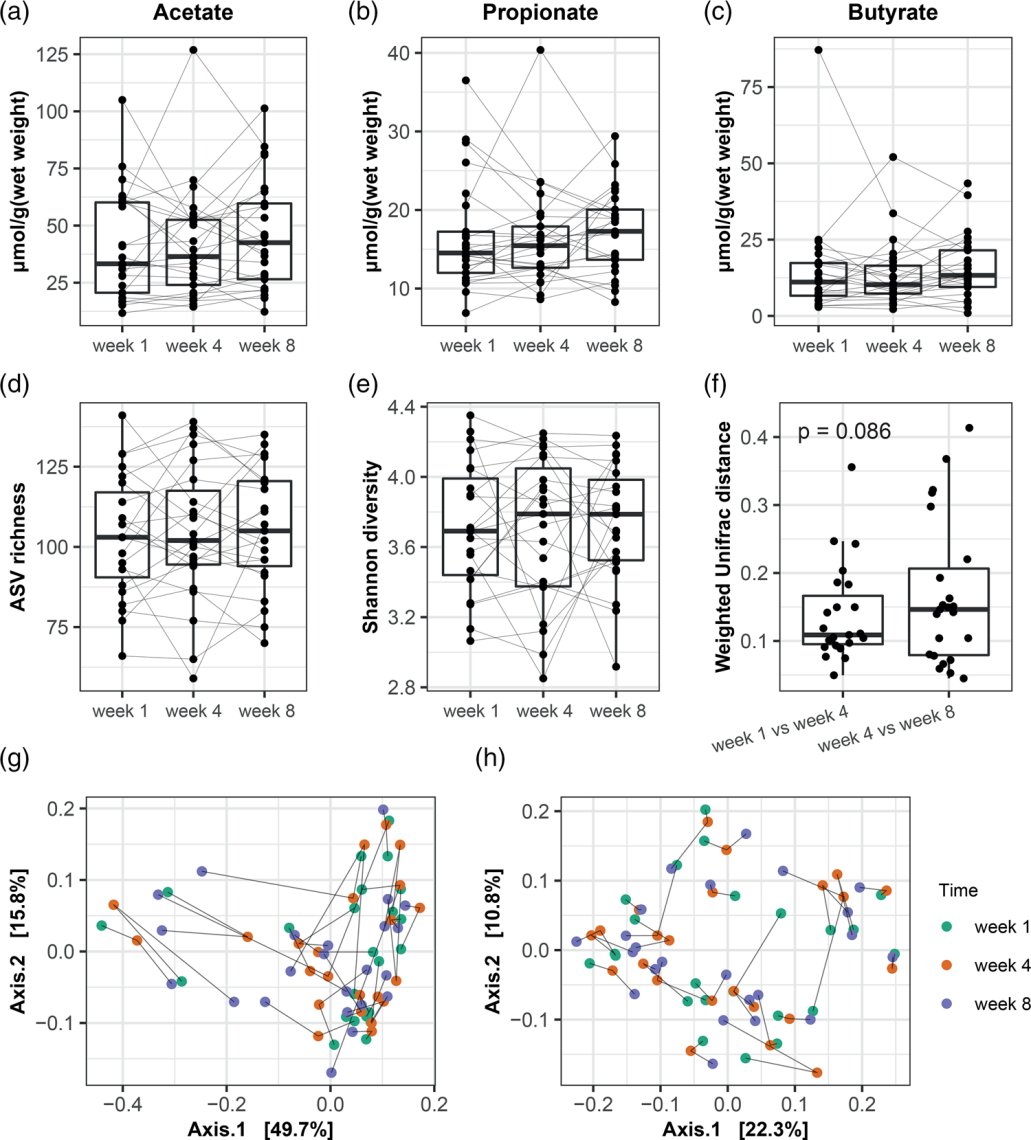
Analysis of short-chain fatty acids and faecal microbiota composition over time. **Legend:** Values were presented as interquartile with the boxplot. Samples taken at different timepoints are connected by solid lines per subject. Weeks 1 and 4 were observational, and week 8 is after the intervention. No differences were observed in faecal acetate (a), propionate (b) and butyrate (c), microbiota ASV richness (d) and Shannon diversity (e) between the time points before and after intervention. A trend was observed for the comparison of microbiota composition stability based on weighted Unifrac distances between week 1 *v.* week 4, and week 4 *v.* week 8 (f). PCoA of microbiota composition based on weighted Unifrac distances (g) and unweighted Unifrac distances (h), stratification based on sampling timepoints.

A large variation in acetate (Fig. 6(a)), propionate (Fig. 6(b)) and butyrate (Fig. 6(c)) was observed over time. Median levels of SCFA increased at week 8 compared to week 1 or 4, albeit non-significant. Microbial alpha diversity as shown by ASV richness (Fig. 6(d)) and Shannon diversity (Fig. 6(e)) did not change over time. PCoA based on weighted (Fig. 6(g)) and unweighted (Fig. 6(h)) Unifrac distance indicated no clear separation in microbiota composition before and after the intervention. However, microbiota composition distance over time tended to be higher between weeks 4 and 8 as compared to weeks 1 and 4, indicating that the composition changed more during the intervention period than during the observation period (Fig. 6(f), *P* = 0·086).

Mixed model analysis showed no effect over time of dietary fibre on acetate (*β* = 0·45 (−0·24; 1·14), *P* = 0·197), propionate (*β* = 0·04 (−0·13; 0·21), *P* = 0·649) or butyrate (*β* = 0·17 (−0·14; 0·49), *P* = 0·281). Total constipation severity was borderline significantly associated with all three SCFA over time (Supplementary Table S2), and an increase in the severity of stool complaints was significantly associated with lower levels of all SCFA. The total QoL was borderline significantly associated with propionate and butyrate. For the QoL subscales, an increase in worries and concerns was significantly associated with lower propionate levels (*P* = 0·036), while an increase in physical discomfort was significantly associated with lower butyrate levels (*P* = 0·038).

### Responder/non-responder analysis

Based on the MID of the PAC-SYM, we identified nine responders and sixteen non-responders. All responders were female, and age and BMI were similar between the two groups (age responder = 35·0  (15·0) years and non-responder = 35·0  (12·9) years; BMI responder = 23·4  (2·5) kg/m^2^ and non-responder = 22·4  (2·0) kg/m^2^). Although non-significant, responders had a lower energy intake (1843  (308) *v.* 2158  (476) kcal, *P* = 0·089) and a higher fibre intake (14·2  (5·0) *v.* 12·4  (3·1) g/1000 kcal, *P* = 0·279). Furthermore, responders had a larger change in fibre intake, both in grams (7·2  (7·8) *v.* 4·8  (6·1), *P* = 0·405) and per 1000 kcal (2·64  (4·8) *v.* 0·82  (1·9), *P* = 0·302). No differences were observed for water intake, total physical activity score, faecal microbiota or SCFA.

### The PDA resulted in more knowledge and outcome beliefs, and was well-accepted

Participants’ self-regulation and subjective health regarding diet (i.e. how healthy participants find their own diet) was significantly lower at week 4, but similar at weeks 1 and 8 (Supplementary Table S3). Participants’ subjective knowledge (*P* < 0·001) and outcome beliefs (*P* = 0·036) regarding fibres significantly increased at week 8 (4·92  (1·0); 5·17  (1·1)) compared to week 4 (3·28  (1·3); 4·78 (1·0)). Moreover, participants’ intention to eat more fibres significantly increased at week 8 (5·8  (1·22)) compared to week 1 (4·28 (1·3), *P* < 0·001), but not compared to week 4 (5·41  (1·3), *P* = 0·106). Participants’ subjective health (i.e. how healthy participants find themselves) did not significantly change between the different measurement moments (week 1 = 5·08  (1·1), week 4 = 4·76  (0·7) and week 8 = 4·84  (0·9)).

Participants rated the PDA on a 7-point Likert scale as positive (5·6  (1·1)), useful (5·6  (1·3)), attractive (5·0  (1·4)) and interesting (5·3  (1·4)). Furthermore, participants positively evaluated the PDA regarding the following aspects: motivational to make high-fibre choices (6·0  (0·9)), help to sustain these changes in dietary intake for long term (6·0  (0·9)), provide insights in their own fibre intake (6·6  (0·8)) and how to improve their fibre intake (6·3  (1·0)), and even though the score was slightly lower, actually improving their fibre intake (5·8  (1·0)).

## Discussion

This study showed that PDA was effective in increasing dietary fibre intake and subsequently improving constipation severity and QoL. Moreover, we observed that an increased fibre intake was associated with the reduction in mild constipation complaints, which remained when adjusted for physical activity and water intake. Although stool frequency did not change, stool consistency softened during the intervention. Faecal microbiota and SCFA do not change significantly, but we showed an association between SCFA and several subscales of constipation severity and QoL. Questionnaires revealed that the PDA increased subjective knowledge and outcome beliefs and was well-accepted.

Our study was the first to use the PDA to improve mild constipation complaints in adults. To our knowledge, only a few studies have used a high-fibre diet instead of fibre supplements to improve symptoms. A study from Anti *et al.* showed that a fibre intake of ≥25 g/d significantly increased stool frequency^(25)^, which we did not observe. This discrepancy might be explained by the magnitude of the change in fibre intake: even though our endpoint was similar, their baseline fibre intake was much lower at approximately 13 g/d, therefore having a larger window of opportunity. We also saw a bigger change in fibre intake in responders. As compared to our previous high-fibre PDA intervention in healthy adults^(35)^, a bigger change in fibre intake was achieved in this study. Possibly, adults with complaints were more motivated which resulted in more substantial changes. Furthermore, we optimised the PDA (e.g. user-friendliness, more high-fibre alternatives), and in contrast to the previous study, fibre intake was now attentively assessed before the start of the intervention.

Several meta-analyses have been done regarding fibre supplementation in constipation and has been shown to be effective in improving symptoms^(14,71,72)^. However, study populations vary greatly, as the Rome criteria for constipation are far from optimal^(73,74)^, which is reflected in low-quality evidence from these trials and large differences in response rates^(14,71,72)^. Fibre supplementations ranged between 10 and 22·5 g/d, which was higher than the change we achieved via the diet. However, there are substantial benefits from increasing fibre intake via the diet. By increasing the intake of healthy foods such as fruits, vegetables, whole grain and legumes, not only positive effects on constipation complaints but also other health effects can be achieved. A high fruit, vegetable, legume and nut intake can reduce the risk of, for example, coronary heart disease^(75–77)^ and obesity^(78–80)^, and does not only provide fibres but also other essential nutrients. In our study, whole grain bread/crispbreads and fruit intake were significantly higher after the PDA. Therefore, even though current guidelines do not distinguish between an fibre increase via diet or supplements^(42)^, the present results suggest that it would be beneficial and feasible for mild constipation complaints and overall health to start with dietary adjustments. Furthermore, spreading fibre intake throughout the day and gradually increasing intake improve tolerability and can prevent additional bloating and cramps that can coincide with an increased fibre intake^(26)^.

Contradicting previous research, we did not observe a significant change in faecal microbiota or SCFA and no associations with fibre intake^(21,81,82)^. However, we did observe a larger change in microbiota distance during the intervention period. Possibly, the change in fibre intake and overall diet was too small to instigate distinct changes, which needs to be larger to be reflected in the stool. Another explanation is the participant-specificity of both microbiota and change in fibre consumption (amount as well as type) making a uniform microbiota change unlikely. Furthermore, 80–95 % of the SCFA are estimated to be absorbed in the gut^(83,84)^, which can mask the possible effects of an increased fibre intake on the SCFA production. We observed an association between all SCFA and severity of stool complaints, between butyrate and physical discomfort, and between propionate and worries and concerns over time. Supporting the present results, faecal SCFA production has been associated with constipation severity before and was shown to be lower compared to healthy adults^(85)^. Butyrate is known for its anti-inflammatory properties and reduction of oxidative stress in the gut and has the ability to reduce visceral sensitivity^(86,87)^. Propionate has been suggested to have a beneficial effect on the blood brain barrier *in vitro*, suggesting a link with mental well-being^(88)^. However, much of the physiology remains unknown and needs further research.

The adults included in this trial had mainly mild symptoms, which was confirmed by the baseline severity score of 1·45  (0·7), which is lower compared to other studies which reported a score ranging between 1·91 and 2·85^(46,89,90)^. We chose to target a population with mild constipation complaints as we expected the largest benefit from a dietary intervention in this group. The average change in the severity score was 0·49  (0·49), which is lower than the clinical relevant change threshold of 0·6^(67)^. This might be due to the more mild symptoms and therefore having a smaller window of opportunity. However, despite the fact that this group mainly had mild symptoms, we still achieved a clinical relevant improvement in 36 % of the study population, and we did see moderate to strong effect sizes for QoL scores^(48)^, and a clear link with dietary fibre intake. This shows that the present results are promising, and highlights the need for future studies with dietary interventions in a population with more severe symptoms.

An important limitation of our study is the lack of a proper placebo group. In patients with abdominal complaints, especially in IBS, the placebo effect has been well-described^(91–93)^. Since it was impossible to include a proper placebo group, a possible placebo effect or regression to the mean effect could have been present, which might drive the improvements in symptoms and the QoL. However, a more objective measure such as stool consistency also significantly improved. Furthermore, the observation period was designed to correct for time or study effects. A cross-over design was not possible due to the nature of the intervention, and including a proper placebo group is difficult in studies with dietary advice and not optimal in this population due to the large between-person variability^(36,37)^. Moreover, fibre intake significantly increased which aids to a healthy lifestyle. Therefore, it can be debated whether a placebo effect is a problem, or if such an intervention positively influencing diet and complaints is helpful, regardless of a possible placebo effect.

Our study is strengthened by the amplitude of measurements, which aids to a more complete overview of the mildly constipated adult, including faecal material, and dietary, physical activity and behavioural assessments. Furthermore, by following participants for 4 weeks without an intervention, we were able to obtain an accurate baseline taking within person variation into account. The use of daily EMA questions increased the accuracy of our measurements, as records have shown to overreport pain and stool frequency compared to EMA in IBS patients^(50)^. With our study design, we were able to capture the daily variation in stool pattern and abdominal pain over time. Furthermore, we used a validated method to obtain dietary data, and included several days to take variation into account^(53)^, which aids to estimate dietary intake more correctly.

In conclusion, our study showed that a digital PDA to increase fibre intake was effective and subsequently improved mild constipation complaints and the QoL. Faecal SCFA was not associated with fibre intake but was with constipation complaints and QoL. The PDA was well-accepted by study participants. The present results indicate that increasing dietary fibre intake via dietary adjustments might be a well-effective first step in the treatment of mild constipation complaints. Future research is needed to assess the effects of dietary adjustments in adults with constipation complaints on a larger scale and in a more severely constipated population. Furthermore, the long-term efficacy and feasibility of PDA needs to be explored.

## Supplementary material

For supplementary material accompanying this paper visit https://doi.org/10.1017/jns.2022.27. click here to view supplementary material
