# Supplementary material for: A high-fibre personalised dietary advice given via a web tool reduces constipation complaints in adults
Source: J Nutr Sci. 2022 Apr 28;11:e31. doi: 10.1017/jns.2022.27 (PMC9066321; doi:10.1017/jns.2022.27)
Supplement: Supplementary file 1 [file jnssup.zip › S2048679022000271sup002.pdf]

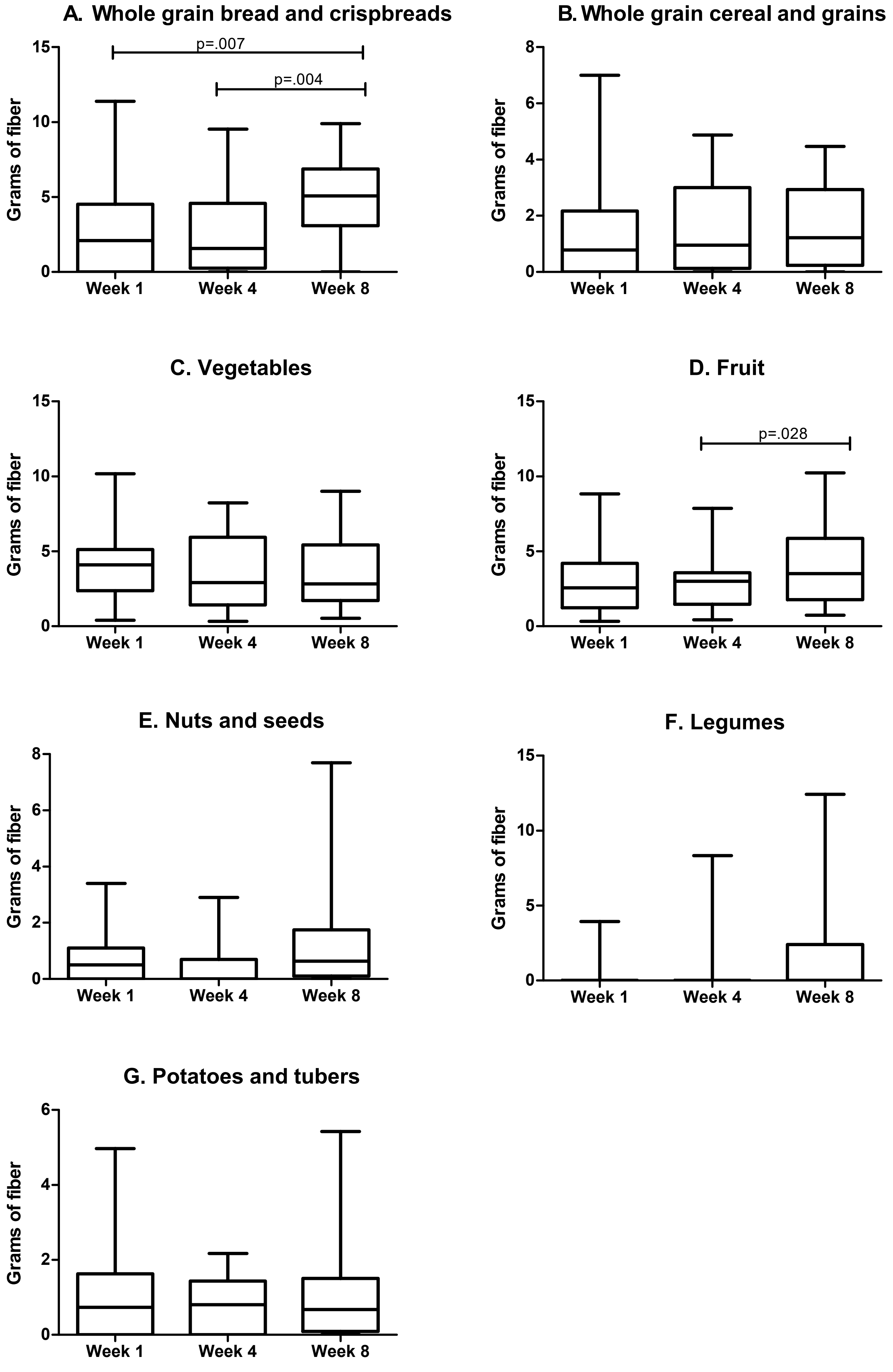
A. Whole grain bread and crispbreads
p=.007
p=.004
Grams of fiber
15
10
5
0
Week 1
Week 4
Week 8
B. Whole grain cereal and grains
Grams of fiber
8
6
4
2
0
Week 1
Week 4
Week 8
C. Vegetables
Grams of fiber
15
10
5
0
Week 1
Week 4
Week 8
D. Fruit
p=.028
Grams of fiber
15
10
5
0
Week 1
Week 4
Week 8
E. Nuts and seeds
Grams of fiber
8
6
4
2
0
Week 1
Week 4
Week 8
F. Legumes
Grams of fiber
15
10
5
0
Week 1
Week 4
Week 8
G. Potatoes and tubers
Grams of fiber
6
4
2
0
Week 1
Week 4
Week 8
