# Supplementary figures and images for: A high-fibre personalised dietary advice given via a web tool reduces constipation complaints in adults
Source: J Nutr Sci. 2022 Apr 28;11:e31. doi: 10.1017/jns.2022.27 (PMC9066321; doi:10.1017/jns.2022.27)

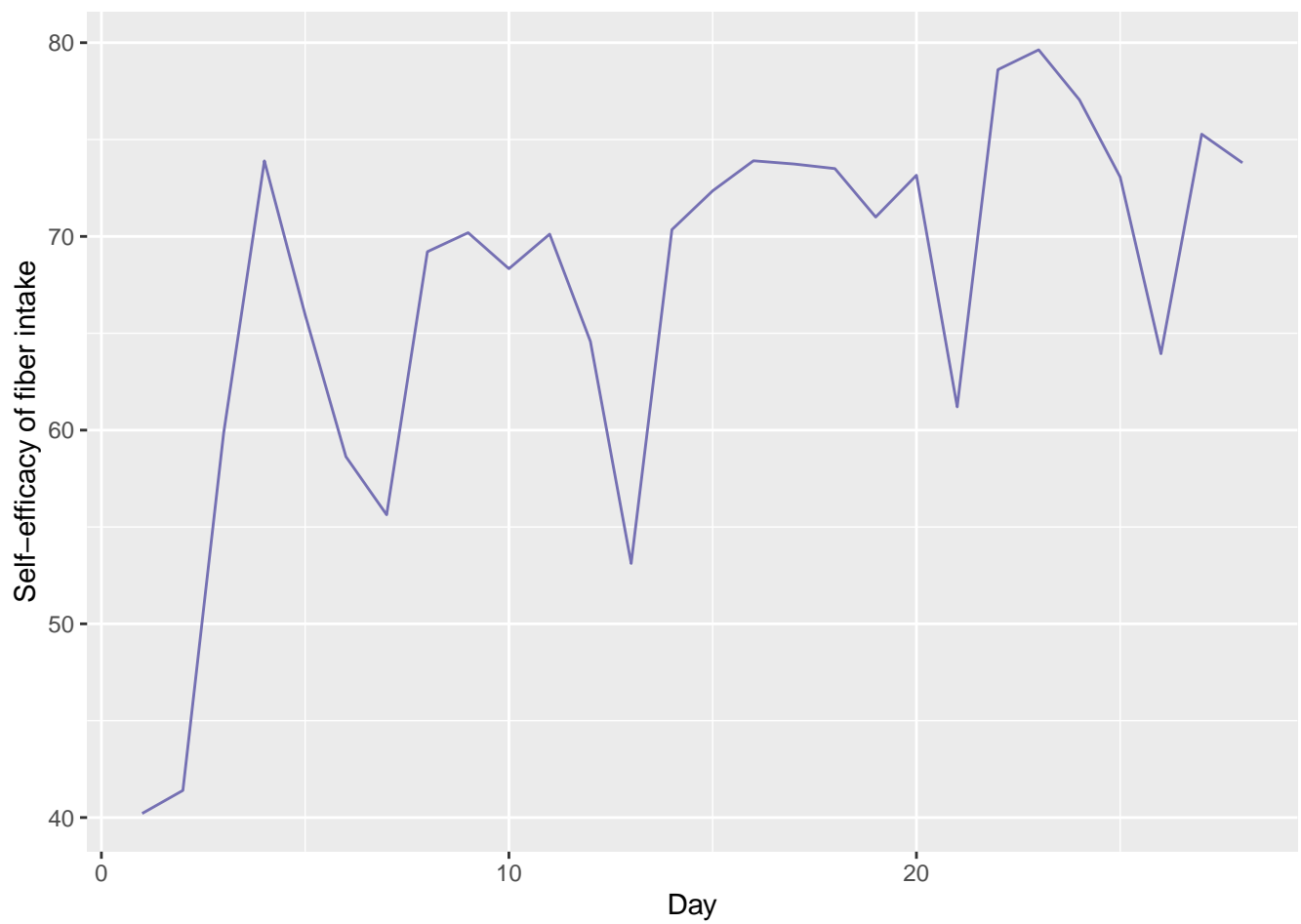

Supplement: Supplementary file 1 [file jnssup.zip › S2048679022000271sup001.pdf]
